# Factors influencing pain medication and opioid use in patients with musculoskeletal injuries: a retrospective insurance claims database study

**DOI:** 10.1038/s41598-024-52477-7

**Published:** 2024-01-23

**Authors:** Stefan Markus Scholz, Nicolas Fabrice Thalmann, Dominic Müller, Maurizio Alen Trippolini, Maria Monika Wertli

**Affiliations:** 1 Department of Statistics, Suva (Swiss National Accident Insurance Fund), Lucerne, Switzerland; 2 Department of General Internal Medicine, University Hospital of Bern, Inselspital, Freiburgstrasse 18, 3010 Bern, Switzerland; 3 School of Health Professions, Bern University of Applied Sciences, Murtenstrasse 10, 3008 Bern, Switzerland; 4 Institute of Physiotherapy, University Hospital of Bern, Inselspital, Freiburgstrasse 18, 3010 Bern, Switzerland; 5 Evidence-Based Insurance Medicine (EbIM), Division of Clinical Epidemiology, Department of Clinical Research, University Hospital Basel, University of Basel, Totengässlein 3, 4051 Basel, Switzerland; 6 Department of Internal Medicine, Kantonsspital Baden, Baden, Switzerland

**Keywords:** Drug development, Epidemiology

## Abstract

Opioid use is only recommended in selected cases of musculoskeletal (MSK) injuries. We assessed factors associated with increased opioid use in MSK injuries. In a retrospective analysis of over four million workers with MSK injuries using the Swiss National Accident Insurance Fund (Suva) database, we analyzed risk factors by multivariate logistic regression. Injury severity was associated with pain medication, opioid, and strong opioid use. Whereas fractures, contusions, and ruptures had higher odds for any pain medication use, increased odds for strong opioids were observed in fractures, superficial injuries, and other injuries. Injuries of the shoulders, elbow, chest, back/spine, thorax, and pelvis/hips showed high odds for opioid use (odds ratio (OR) > 2.0). Injuries of the shoulders had higher odds for strong opioid use (OR 1.136; 95% CI 1.040–1.241). The odds for using strong opioids increased from 2008 OR 0.843 (95% confidence interval (CI) 0.798–0.891) to 2018 OR 1.503 (95% CI 1.431–1.578), compared to 2013. Injury severity, type of injury, and injured body parts influenced the use of pain medication and overall opioid use in musculoskeletal injuries. Strong opioids were more often used in fractures but also in superficial and other minor injuries, which indicates that other factors play a role when prescribing strong opioids.

## Introduction

In the U.S. an opioid crisis resulted in an opioid addiction epidemic and decreased life-expectancy^1–3^. Whereas in the U.S. the use of prescribed opioids decreased between 2010 and 2017^4,5^, there is an ongoing controversy in Europe whether currently an opioid crisis is unfolding^6–8^. Several countries report an increased use of opioids^9–13^. Thus, an increased use of opioids for non-cancer pain is of growing concern also in Europe. Research about the clinical implications is rare and many studies use indirect evidence to assess the potential implications of increased opioid use^9,12,13^. Increased use in opioids may be due to improved palliative care, better access to opioids, and other factors. However, a recent study observed an increase in opioid related poisoning indicating that at least in some countries opioid use may have reached problematic proportions^14^.

In order to address potential under and overuse of opioids, studies are needed to understand how prescription practices changed over time and factors that may influence the decision process. Musculoskeletal (MSK) injuries are major contributors to chronic pain^15–18^. In MSK injuries, studies consistently showed that strong opioids resulted only in a small pain reduction and functional improvement compared to placebo and were no more effective than non-steroidal anti-inflammatory drugs (NSAIDs)^19–21^. Further, opioid use after MSK injuries may be potentially related with unwanted consequences such as delayed recovery^22–24^, disability^25^, adverse effects such as cognitive impairment, hyperalgesia, nausea, and opioid dependence^26,27^. Therefore, guidelines recommend in acute MSK injuries non-opioid analgesic as first choice and restrict weak and strong opioids to patients with otherwise uncontrolled pain and severe injuries^28,29^. Thus, analyzing medication prescription patterns in MSK injuries may enhance our understanding about potential influencing factors.

A study in the primary care setting in the United Kingdom showed that 59% of patients with chronic MSK pain received opioid prescriptions in 2011/2012^30^. In Switzerland, the use in strong opioids increased substantially over time^12,31^. Using claims data, we observed an increase in strong opioid use in minor and major MSK injuries confirming a more liberal prescription practice of opioids even in injuries where non-opioids are the preferred choice^31^. However, we observed considerable unexplained differences across Swiss cantons (administrative regions). Large variation in care across geographically close areas are often attributed to differences in practice style and physician preferences^32^. One criticism of studies assessing variation in care is, that other factors such as type of injury and severity of injury may explain the observed variation. To date only limited information about the clinical factors that influence pain medication use in a national cohort of workers with MSK injuries is available. Pain medication use may be influenced by injury severity, type of injury, or location of injuries thus, explaining variation in pain medication use in studies.

Therefore, the aim of the current study was to assess potential patient and injury-related factors that may be associated with pain medication and opioid use. We analyzed all MSK injuries in a representative cohort of Swiss workers between 2008 and 2018. In this study, we addressed three questions: (1) Which case properties are making it more probable that pain medications are used for the treatment of a musculoskeletal injury? (2) Amongst those cases treated with pain medications, which case properties are making it more probable that opioids are used? (3) Amongst those cases treated with opioids, which case properties are making it more probable that strong opioids are used?

## Methods

The methods used in this study have been previously described^31^.

### Study design

The study cohort included workers with MSK injuries recorded in the Swiss National Accident Insurance Fund (Suva) database. The study was conducted following the International Society for Pharmacoeconomics and Outcomes Research (ISPOR) checklist for retrospective database studies^33^.

### Study population

The study setting and the methods we used have been described in a further study^31^. Briefly, we used insurance claims data from the Suva, the largest accident insurer in the country covering approximately two million people (i.e., 37% of the working population of Switzerland). In Switzerland all employees and all unemployed persons are covered by a compulsory accidence insurance according to the Swiss Accident Insurance Act. This insurance covers costs (wage compensations during work incapacities, long-term disability pensions, medical treatment costs, and other medical expenses) of occupational and non-occupational accidents and occupational diseases.

All MSK injury claims registered at Suva in 2008 to 2018 were included. MSK injuries were identified by injury codes and the affected body parts. We excluded claims for amputations, burns, poisons, and chemical burns, injuries of the respiratory and internal organs, and claims for loss of sexual organs/reproductive ability. We also excluded claims for injuries that resulted in tetra- or paraplegia, injuries of teeth, eye, ear, superficial abrasions, physical shock (e.g., cardiovascular, anaphylactic, electrical shock) and mental shock and traumas (e.g., acute stress disorders), and fatal injuries. Furthermore, we excluded all patients who declined the use of their data for research for data privacy reasons. Finally, we excluded injuries where the injured body part or type of injury was unknown and claims of persons treated outside of Switzerland. Exclusion criteria were based on injured body part and injury type from the accident claims form, or based on insurance administrative data (tetra-and paraplegia, fatalities, data privacy).

### Data sources

Administrative data from the injury claims forms were used to extract sociodemographic information (gender, age at the time of the accident), the year of the accident registration, accident-related information (self-reported injured part of body, type of injury, and accident at work or during leisure time). In patients with multiple claims during the study period, each claim was studied as an independent case.

Data on healthcare expenses for pain medication were retrieved from the administrative Suva database on healthcare costs, which is fed directly from the electronic billing systems. Data were only available for outpatient treatments (billing for inpatient treatments is based on diagnosis related groups). All costs are attributed to a related case. It comprises data by granularity of invoice line items, with either pharmacode or global trade item number (GTIN) code, descriptive text, date, quantities, and invoiced amount of the line item. Pharmacode, GTIN code, and descriptive text were used to identify pain medication.

### Follow-up duration

After registration, each claim was followed-up for two years (730 days). The follow-up duration was chosen to have sufficient follow-up to calculation treatment duration and daily dose of pain medications. All outpatient medication use was included in this analysis.

### Dependent variables

#### Pain medication use

The use of pain medication was assessed during the first 730 days after the date of the injury by identifying the appropriate WHO ATC codes. The WHO ATC/DDD system allows standardization of drug groupings and a stable drug utilization metric to enable comparisons of drug use between countries^34^. Pain medication use included all groups of pain medications: non-opioid pain medications, weak opioids, and strong opioids. Opioid use within a drug substitution program (i.e., diamorphine N07BC06 Diaphin®) was not included and is usually also not reimbursed by the accident insurance.

Non-opioid pain medications included: paracetamol (ATC codes N02BE01, N02BE51), non-steroidal anti-inflammatory drugs (NSAIDs, M01AA, M01AB, M01AC, M01AE, M01AG, M01AX), coxibs (COX-2-inhibitors, M01AH), metamizole (N02BB02, N02BB52).

Weak opioids (defined as opioid formulations with a morphine conversion factor of ≤ 0.3) included dihydrocodeine (N02AA08), codeine (N02AA59, N02AJ06), tilidine (N02AX01), tramadol (N02AX02, N02AX52, N02AJ13), and tapentadol (N02AX06)^35^.

Strong opioids (defined as all other opioids) included morphine (N02AA01), hydromorphone (N02AA03), nicomorphine (N02AA04), oxycodone (N02AA05, 02AA55), pethidine (N02AB02), fentanyl (N02AB03), buprenorphine (N02AE01, N07BC01), nalbuphine (N02AF02), and methadone (N07BC02).

### Independent variables

*Sociodemographic information*: Gender, age at the time of the accident.

*Accident-related information*: Year of the accident registration, accident at work or during leisure time.

*Injury severity*: Accidents were divided into minor cases (≤ 3 days absence from work) and major cases with daily allowances (which are paid when absence from work is more than 3 days).

*Type of injury*: Injury types as indicated on the accident claims form by the employer were categorized into six groups: Fractures, sprain (dislocation, sprain, and strain), rupture (rupture and tear), contusion (contusion and bruises), superficial (superficial injuries and cuts), and other (bites, foreign bodies, inflammation, edema, and bullet wound).

### Statistical analysis

For the descriptive statistics, mean and standard deviation of the victims age was calculated. We used binary logistic regression to identify and quantify the influence of variables. To address the three research questions, three models were created:

1. For all cases in the study, with the use of ≥ 1 pain medication (non-opioid, weak opioid, and/ or strong opioid) as dependent variable.
2. For all cases using pain medications, with the use of opioids (weak and/ or strong opioids) as dependent variable.
3. For all cases using opioids, with the use of strong opioids as dependent variable.

We included age, gender, type of injury, injury severity, year of registration as independent variables in all models. The predictor variables were entered stepwise into the models in forward selection mode with a significance level of 0·05, which was met by all the independent variables. A reference point was designated for every variable (reference age 40 years, and reference year of registration in 2013). Results were reported in odds ratios (OR) and 95% confidence intervals (95% CI). We visualized the OR for the three models using a body chart. Not included in the visualization were the ORs for multiple upper extremity, multiple lower extremity injuries, and multiple injuries of various body parts (polyblessée).

As a sensitivity analysis, we assessed potential interactions between injured body part and type of injury. We grouped the affected body parts into six groups: Head (face and head), upper torso (neck, spine, back, thorax), lower torso (hips, coccyx, thigh, pelvis), upper extremities (shoulder to fingers), lower extremities (upper leg to toes), and other injuries (injuries which could not be assigned the above-mentioned body parts, like mental shock, whole body or systemic effects, injuries to multiple body parts (polyblessées), and other injuries).

We repeated the models replacing the two separate body part and injury type variables by the combined grouped body part × injury type variable as independent.

SAS statistical analysis software version 9·3 (SAS Institute Inc., Cary, NC, USA) was used for statistical analysis throughout the study. The body chart was visualized using the Python 3·7 and the vedo package^36^.

### Ethical approval

The study was approved by the Kantonale Ethikkomission (KEK) Bern number 2020-00718, approved November 2020. Due to the retrospective nature of the study, the need of informed consent was waived by the KEK. The study was conducted in accordance with the declaration of Helsinki and in compliance with the Federal Law of Human Research.

## Results

**Figure 1:**
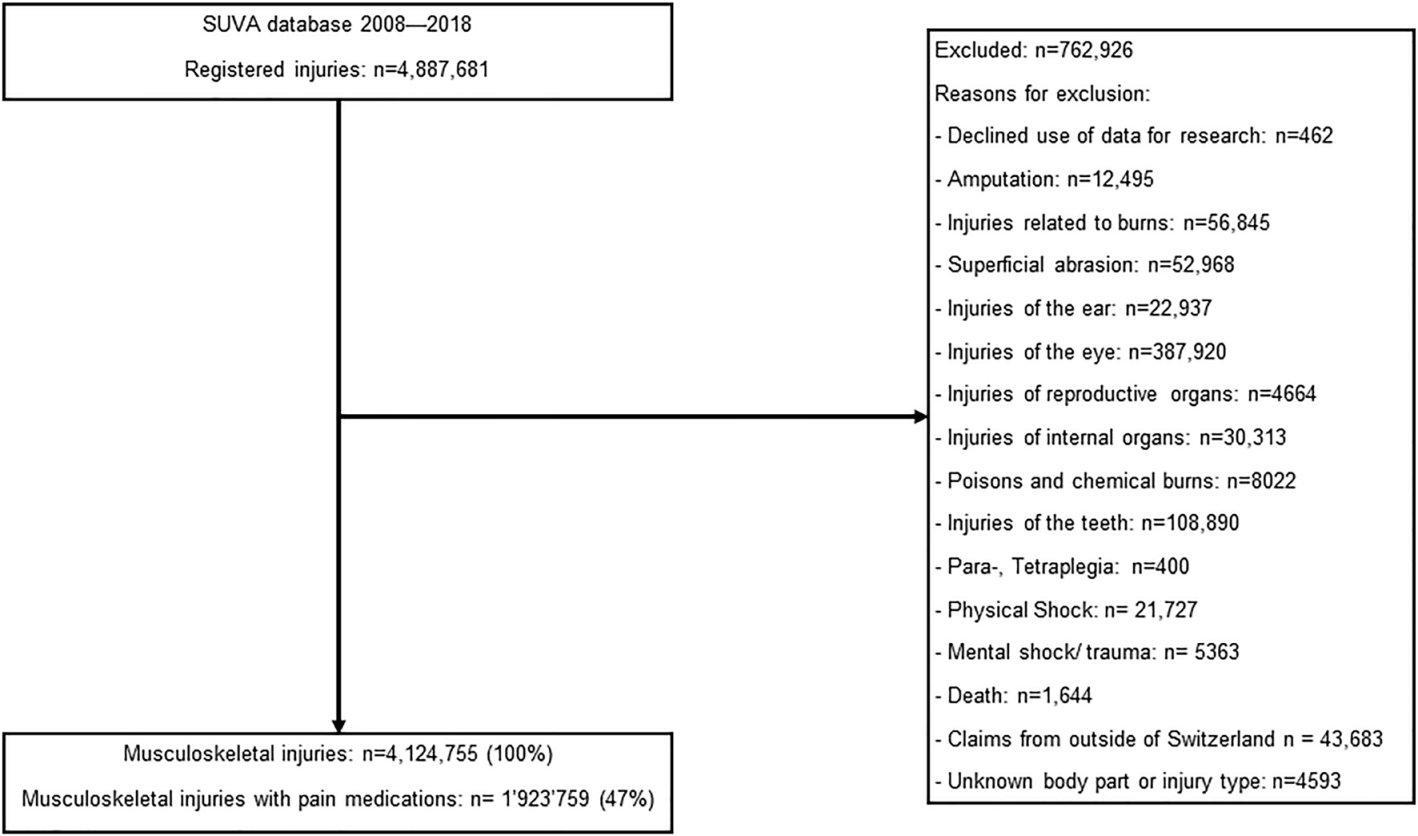
Study flow.

In total, 4,124,755 MSK injuries were recorded between 2008 and 2018 and were included in the study cohort (Fig. 1). Of these cases, 1,923,759 received pain medications (46.6%). Of cases with ≥ 1 pain medication, opioids were used in 227,317 injuries (11.8% weak or strong opioids) and 43,610 cases received strong opioids (2.3%).

Baseline characteristics are given in Table 1. The mean age of the patients was 37.3 years, 78.9% were male, 53.6% were major injuries: Most of the injuries were contusions (25.6%). In injuries with opioid use, the mean age was higher (41.1 years), and the proportion of major injuries higher (88.6%).

**Table 1:** Baseline characteristics.

	All injuries	Injuries with pain medication	Injuries with opioids
	N (%)/mean [SD]		
All injuries	4,124,755 (100)	1,923,759 (46.6)	227,317 (5.5)
Age (years)	37.3 [13.5]	37.8 [13.5]	41.1 [12.9]
Male sex	3,254,221 (78.9)	1,528,404 (79.5)	182,150 (80.1)
Year of injury			
2008	368,845 (8.9)	163,333 (8.5)	18,191 (8.0)
2009	368,971 (8.9)	166,755 (8.7)	19,331 (8.5)
2010	376,736 (9.1)	174,464 (9.1)	20,825 (9.2)
2011	379,714 (9.2)	174,240 (9.1)	20,278 (8.9)
2012	374,695 (9.1)	176,663 (9.2)	20,789 (9.1)
2013	375,491 (9.1)	178,510 (9.3)	21,333 (9.4)
2014	370,790 (9.0)	178,741 (9.3)	20,697 (9.1)
2015	372,693 (9.0)	177,351 (9.2)	21,077 (9.3)
2016	371,780 (9.0)	175,714 (9.1)	20,785 (9.1)
2017	377,736 (9.2)	179,333 (9.3)	22,126 (9.7)
2018	387,304 (9.4)	178,655 (9.3)	21,885 (9.6)
Injury severity			
Minor^§^	1,913,626 (46.4)	589,615 (30.6)	25,911 (11.4)
Major^#^	2,211,129 (53.6)	1,334,144 (69.4)	201,406 (88.6)
Injury type			
Fracture	376,077 (9.1)	243,857 (12.7)	55,107 (24.2)
Contusion	1,055,991 (25.6)	530,442 (27.6)	59,046 (26.0)
Rupture	305,671 (7.4)	162,145 (8.4)	17,638 (7.8)
Superficial	620,426 (15.0)	168,958 (8.8)	9968 (4.4)
Sprain	798,809 (19.4)	396,657 (20.6)	35,670 (15.7)
Other	967,781 (23.5)	421,700 (21.9)	49,888 (21.9)
Injured body parts			
Head	161,790 (3.9)	56,852 (3.0)	4048 (1.8)
Face	148,314 (3.6)	44,454 (2.3)	2634(1.2)
Neck	36,474 (0.9)	14,485 (0.8)	1468 (0.6)
Back	193,291 (4.7)	109,381 (5.7)	25,433 (11.2)
Spine	104,346 (2.5)	57,712 (3.0)	10,245 (4.5)
Thorax	165,881 (4.0)	105,762 (5.5)	29,011 (12.8)
Shoulder	277,956 (6.7)	171,069 (8.9)	35,923 (15.8)
Upper arm	71,272 (1.7)	32,799 (1.7)	5741 (2.5)
Elbow	91,614 (2.2)	45,563 (2.4)	4254 (1.9)
Lower arm	76,631 (1.9)	30,170 (1.6)	2816 (1.2)
Wrist	206,195 (5.0)	95,038 (4.9)	9104 (4.0)
Hand	199,109 (4.8)	74,051 (3.8)	5734 (2.5)
Finger	666,327 (16.2)	236,043 (12.3)	16,864 (7.4)
Pelvis	20,598 (0.5)	11,569 (0.6)	2451 (1.1)
Hips	25,957 (0.6)	13,586 (0.7)	2165 (1.0)
Coccyx	27,411 (0.7)	15,338 (0.8)	3147 (1.4)
Thigh	29,263 (0.7)	9546 (0.5)	913 (0.4)
Upper leg	94,713 (2.3)	37,392 (1.9)	3316 (1.5)
Knee	485,609 (11.8)	268,458 (14.0)	26,336 (11.6)
Lower leg	171,635 (4.2)	72,955 (3.8)	7924 (3.5)
Ankle	496,151 (12.0)	251,201 (13.1)	13,935 (6.1)
Foot	182,101 (4.4)	86,343 (4.5)	5580 (2.5)
Toes	135,659 (3.3)	53,159 (2.8)	2609 (1.1)
Multiple upper extremity injuries	21,315 (0.5)	11,747 (0.6)	2043 (0.9)
Multiple lower extremity injuries	10,322 (0.3)	5011 (0.3)	694 (0.3)
Multiple injuries (polyblessé)	24,821 (0.6)	14,075 (0.7)	2929 (1.3)

*SD* standard deviation.

^**§**^Minor injury, < 3 days absence from work.

^#^Major injury, 3 or more days absence from work.

### Predictors for pain medication use (model 1)

**Figure 2:**
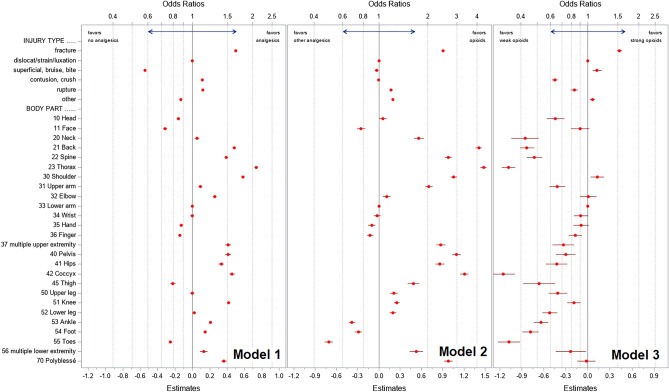
Odds ratios for injury type and affected body part for pain medication, opioid, and strong opioid use in a cohort of Swiss workers. Odds ratios and 95% confidence intervals derived from logistic regression models using all cases for predicting the event “cases treated with any analgesic” (Model 1, left), using cases with analgesics for predicting the event “cases treated with opioid” (Model 2, center), and using cases treated with opioids for predicting the event “cases treated with strong opioid” (Model 3, right).

Older age was associated with more pain medication use. Pain medication use overall decreased over time (OR 0·919 compared to 2013, Table 2). Compared to men, women were less likely to receive pain medications (OR 0.975; 95% CI 0.969–0.980). Minor injuries were less likely to receive pain medications (OR 0.330; 95% CI 0.329–0.331, Table 3, Fig. 2). Fractures, contusion, and rupture were associated with more pain medication use. Further, injuries in multiple locations were associated with more medication use. The highest OR for pain medication use were observed in injuries of the thorax with OR > 2, compared to our reference point, followed by shoulder, back, coccyx, pelvis, spine, and hips. The face and toes are the least likely body parts to be treated with pain medication.

**Table 2:** Odds ratios for pain medication use, opioid use, and strong opioid use in workers with musculoskeletal injuries by baseline predictors.

	OR (95% CI)		
	Model 1*	Model 2^§^	Model 3^#^
Female sex (ref. male)	**0.975 (0.969; 0.980)**	**0.983 (0.972; 0.995)**	**0.933 (0.907; 0.960)**
Age			
20-year point estimate	**0.925 (0.922; 0.928)**	**0.751 (0.746; 0.756)**	**1.163 (1.143; 1.183)**
30	**0.962 (0.960; 0.963)**	**0.867 (0.863; 0.870)**	**1.079 (1.069; 1.088)**
40 (ref)	1.000 (1.000; 1.000)	1.000 (1.000; 1.000)	1.000 (1.000; 1.000)
50	**1.040 (1.038; 1.041)**	**1.154 (1.150; 1.158)**	**0.927 (0.919; 0.935)**
60-year point estimate	**1.081 (1.077; 1.084)**	**1.332 (1.322; 1.341)**	**0.860 (0.845; 0.875)**
Year of injury			
2008	**0.863 (0.855; 0.872)**	**0.926 (0.906; 0.948)**	**0.843 (0.798; 0.891)**
2009	**0.896 (0.887; 0.905)**	**0.961 (0.940; 0.983)**	**0.868 (0.822; 0.916)**
2010	**0.945 (0.936; 0.954)**	1.002 (0.980; 1.024)	**0.881 (0.835; 0.929)**
2011	**0.943 (0.933; 0.952)**	0.992 (0.970; 1.014)	0.966 (0.917; 1.018)
2012	**0.979 (0.970; 0.989)**	0.988 (0.966; 1.010)	0.961 (0.912; 1.012)
2013 (ref)	1.000 (1.000; 1.000)	1.000 (1.000; 1.000)	1.000 (1.000; 1.000)
2014	**1.029 (1.019; 1.040)**	**0.978 (0.957; 0.999)**	**1.086 (1.032; 1.144)**
2015	1.001 (0.991; 1.011)	1.001 (0.980; 1.023)	**1.218 (1.158; 1.281)**
2016	**0.982 (0.973; 0.992)**	0.991 (0.969; 1.013)	**1.278 (1.216; 1.344)**
2017	**0.981 (0.972; 0.991)**	**1.025 (1.003; 1.047)**	**1.373 (1.307; 1.442)**
2018	**0.919 (0.910; 0.928)**	0.996 (0.975; 1.018)	**1.503 (1.431; 1.578)**

*Model 1: For all injuries predicting use of ≥ 1 pain medication (non-opioid, weak opioid, and/or strong opioid) as dependent variable.

^§^Model 2: For all injuries with ≥ 1 pain medication predicting opioid use (weak opioid, and/or strong opioid).

^#^Model 3: For all injuries with opioid use predicting strong opioid use.

*OR* odd ratio, *95% CI* 95% confidence interval; minor injury, less than 3 days allowances; major injury 3 + days allowances.

Bold indicates significant values.

Point estimates are indicated for specific ages between 20 to 60 years.

**Table 3:** Odds ratios for pain medication use, opioid use, and strong opioid use in workers with musculoskeletal injuries by injury related predictors (cont’d from Table 2).

	OR (95% CI)		
	Model 1*	Model 2^§^	Model 3^#^
Injury severity^‡^			
Minor (ref. major)	**0.330 (0.329; 0.331)**	**0.270 (0.266; 0.274)**	**0.417 (0.397; 0.439)**
Injury type			
Fracture	**1.650 (1.634; 1.665)**	**2.468 (2.427; 2.509)**	**1.534 (1.480; 1.591)**
Sprain (ref)	1.000 (1.000; 1.000)	1.000 (1.000; 1.000)	1.000 (1.000; 1.000)
Superficial	**0.581 (0.576; 0.586)**	**0.970 (0.946; 0.996)**	**1.132 (1.065; 1.203)**
Contusion	**1.120 (1.113; 1.128)**	0.994 (0.979; 1.010)	**0.635 (0.611; 0.661)**
Rupture	**1.127 (1.117; 1.137)**	**1.189 (1.165; 1.213)**	**0.830 (0.791; 0.871)**
Other	**0.875 (0.869; 0.881)**	**1.219 (1.201; 1.238)**	**1.062 (1.024; 1.101)**
Injured body parts			
Shoulder	**1.792 (1.760; 1.824)**	**2.877 (2.757; 3.003)**	**1.136 (1.040; 1.241)**
Upper arm	**1.099 (1.074; 1.124)**	**2.027 (1.927; 2.132)**	**0.660 (0.593; 0.735)**
Elbow	**1.294 (1.267; 1.322)**	**1.117 (1.060; 1.177)**	1.003 (0.899; 1.120)
Lower arm (ref)	1.000 (1.000; 1.000)	1.000 (1.000; 1.000)	1.000 (1.000; 1.000)
Wrist	0.998 (0.980; 1.016)	0.976 (0.932; 1.022)	**0.907 (0.823; 0.999)**
Hand	**0.880 (0.863; 0.896)**	**0.902 (0.859; 0.948)**	0.909 (0.820; 1.008)
Finger	**0.866 (0.852; 0.881)**	**0.883 (0.845; 0.923)**	**0.841 (0.767; 0.922)**
Head	**0.852 (0.836; 0.868)**	**1.059 (1.005; 1.116)**	**0.642 (0.569; 0.724)**
Face	**0.731 (0.716; 0.745)**	**0.777 (0.733; 0.823)**	0.898 (0.793; 1.017)
Neck	**1.057 (1.028; 1.086)**	**1.756 (1.638; 1.882)**	**0.424 (0.352; 0.512)**
Back	**1.617 (1.588; 1.648)**	**4.091 (3.917; 4.274)**	**0.434 (0.395; 0.477)**
Spine	**1.480 (1.450; 1.511)**	**2.658 (2.537; 2.786)**	**0.482 (0.435; 0.534)**
Thorax	**2.083 (2.043; 2.123)**	**4.379 (4.193; 4.573)**	**0.339 (0.308; 0.372)**
Pelvis	**1.509 (1.459; 1.560)**	**2.985 (2.804; 3.179)**	**0.739 (0.647; 0.845)**
Coccyx	**1.580 (1.533; 1.628)**	**3.343 (3.153; 3.544)**	**0.315 (0.270; 0.367)**
Hips	**1.394 (1.352; 1.437)**	**2.364 (2.219; 2.519)**	**0.652 (0.563; 0.754)**
Thigh	**0.798 (0.774; 0.823)**	**1.628 (1.499; 1.767)**	**0.513 (0.414; 0.636)**
Upper leg	0.997 (0.976; 1.019)	**1.236 (1.170; 1.306)**	**0.664 (0.585; 0.752)**
Knee	**1.517 (1.491; 1.543)**	**1.286 (1.231; 1.343)**	**0.824 (0.752; 0.902)**
Lower leg	**1.021 (1.002; 1.041)**	**1.216 (1.160; 1.275)**	**0.594 (0.537; 0.658)**
Ankle	**1.229 (1.208; 1.250)**	**0.683 (0.653; 0.714)**	**0.528 (0.479; 0.581)**
Foot	**1.157 (1.135; 1.179)**	**0.747 (0.711; 0.785)**	**0.457 (0.408; 0.512)**
Toes	**0.776 (0.761; 0.792)**	**0.492 (0.464; 0.521)**	**0.341 (0.294; 0.396)**
Multiple upper extremity	**1.512 (1.463; 1.563)**	**2.398 (2.248; 2.558)**	**0.714 (0.618; 0.825)**
Multiple lower extremity	**1.142 (1.092; 1.194)**	**1.692 (1.541; 1.858)**	**0.790 (0.643; 0.971)**
Multiple injuries (polyblessée)	**1.439 (1.395; 1.485)**	**2.661 (2.508; 2.823)**	**0.979 (0.866; 1.106)**

*Model 1: For all injuries predicting use of ≥ 1 pain medication (non-opioid, weak opioid, and/or strong opioid) as dependent variable.

^§^Model 2: For all injuries with ≥ 1 pain medication predicting opioid use (weak opioid, and/or strong opioid).

^#^Model 3: For all injuries with opioid use predicting strong opioid use.

*OR* odds ratio, *95% CI* 95% confidence interval.

Bold indicates significant values.

^‡^injury severity: Minor injury, < 3 days absence from work; major injury, 3 or more days absence from work.

### Predictors for opioid use (model 2)

In all patients receiving pain medications (1,923,759 injuries), older age and male sex was again associated with more opioid use (Table 2). Overall opioid use remained unchanged over time. Minor injuries were less likely to receive opioids (Table 3, Fig. 2). Fractures, and—to a lesser degree—ruptures, and other injuries were associated with more opioid use. More opioid use was observed in injuries of several body parts. The highest OR for opioid use were observed in injuries of the thorax and back, both with OR > 4, compared to our reference point, followed by coccyx, pelvis, shoulder, spine, and hips. Injuries of the ankle, hand, foot, and toes were less likely to receive opioids.

### Predictors for strong opioid use (model 3)

In all patients receiving opioid medications (227,317 injuries), younger patients were more likely to receive strong opioids (OR for the point estimate age 20 years 1.163 (95% CI 1.143–1.183) compared to reference age of 40 years, Table 2). There was an almost linear increase in strong opioid use over time: The 2008 OR was 0.843 (95% CI 0.798–0.891) compared to 2013. The OR for the year 2018 was 1.503 (95% CI 1.431–1.578) compared to 2013. Minor injuries were less likely to receive strong opioids (OR 0.417, 95% CI 0.397–0.439, Table 3, Fig. 2). Strong opioids were more used in fractures (with OR > 1.5) and—to a much lesser degree—for superficial injuries. The highest OR for strong opioid use were observed in injuries of the shoulder, but a low OR for the thorax and other parts of the torso.

### Body chart

**Figure 3:**
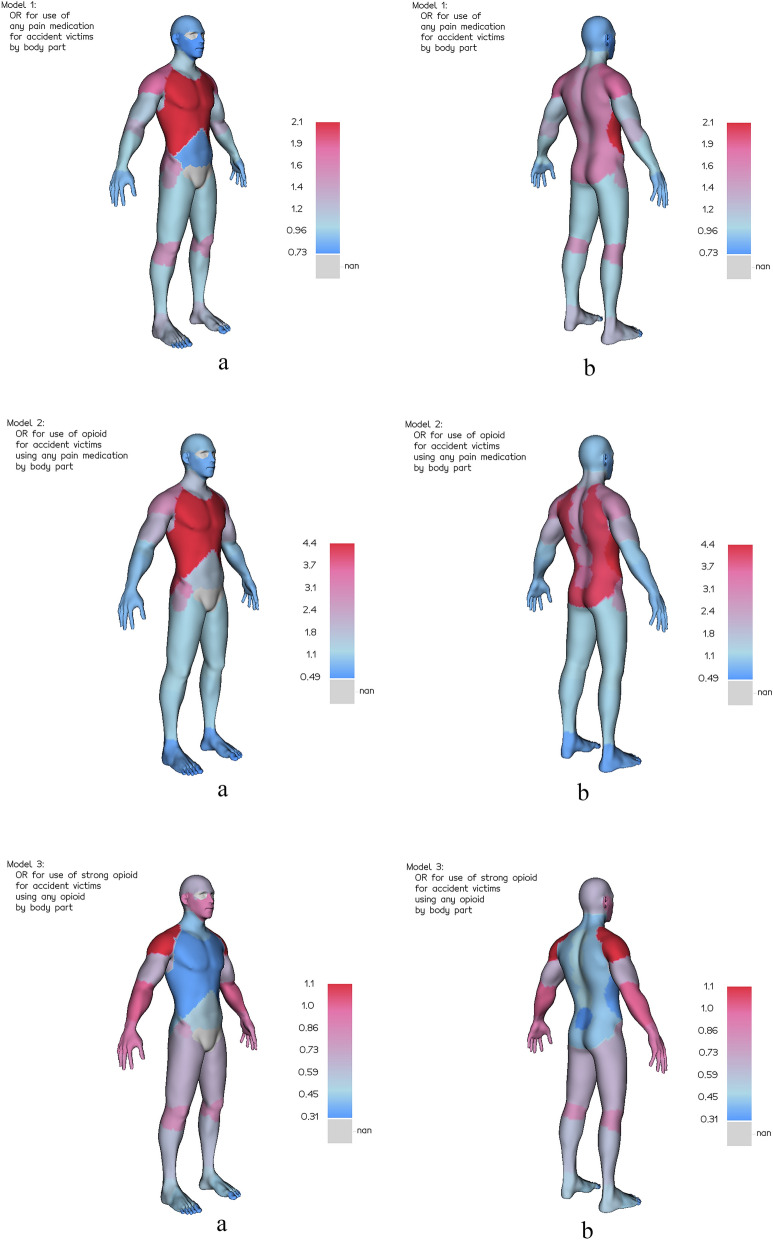
Body chart of the Odds Ratios from Table 3 for pain medication use by location of injury and medication type. Lower arm is set to OR = 1 as reference point. Cases with multiple injured body parts are not shown.

Figure 3 visualizes the odds ratios for pain medication use, opioid use, and strong opioid use by body part. As a general trend, we observed increasing ORs for pain medication starting distal parts of the extremities to the proximal parts. The bigger joints (knee, elbow, shoulder, hips) show increased use of pain medication. The highest OR for pain medication is found for the thorax. A similar pattern with increasing ORs from distal to proximal parts of extremities and the thorax were seen for any opioid use. Injuries to the knee and elbow joints were not associated with an increased OR for the use of opioids. Injuries of the shoulder were the injuries most associated to strong opioid use.

### Sensitivity analysis

In the sensitivity analysis we assessed potential interactions between body part and injury type variables in the same three models. Characteristics of the categories with these interactions are provided in Appendix Table 1. The findings of our models were robust when replacing body part and injury type variable with an interaction between the body part and injury type variable (Appendix Fig. 2). The differences between OR from our models and the models in the sensitivity analysis were small. OR for the interactions between body part and injury type are provided in Appendix Table 1.

## Discussion

In this analysis of more than four million MSK injuries in a cohort of workers, we observed an association between various demographic and injury related factors and the use of pain medications. Whereas the overall use of pain medication decreased over the study period, the use of opioids remained unchanged and there was a linear increase in the use of strong opioids. In major injuries the odds for pain medication, opioid, and strong opioid use increased. The effects of most injury related factors point in the expected direction, such as that fractures or higher injury severity increased the use of pain medications, opioids, and the use of strong opioids. In general, the results indicate a prescribing pattern of pain medication and overall opioid use that increases from more distal to the more proximal parts of the extremities. Bigger joints (knee, elbow, shoulder, hips) and injuries to the torso showed the highest odds. If opioids are used, injuries of the shoulders were the injuries with the highest odds for the use of strong opioids.

Comparable to our findings, other European countries observed an increase in the use of strong opioids^9–13,30^. For example, in the Netherlands opioid prescriptions increased in the total population per year from 4.9% in 2013 to 6.0% in 2017^9^. In four German federal states a similar increase was observed (4.2% in 2006 to 4.9% in 2016^10^). In Switzerland, the use in strong opioids increased substantially over time^12^ and a recently published study confirmed a more liberal prescription of strong opioids in MSK injuries^31^. Further, an increase in opioid related poisonings indicate that at least in some European countries opioid use may have reached problematic proportions^14^.

Despite the increasing evidence of potential harm of opioid use^22–24,26,27,37,38^ and guideline recommendations to restrict opioids to selected patients^39^, up to 87.1% primary care physicians prescribed strong opioids for MSK pain^40^. In the United Kingdom, primary care physicians prescribed opioids in 59% of patients with chronic musculoskeletal pain in 2011/2012^30^. In Australia 23.6% of workers with lower limb injury claims received opioids and opioid use was associated with delayed return to work^41^. In the U.S., the prevalence of prescribed opioids in MSK conditions (back or neck problems, arthritis, rheumatism) increased by 7% from 12% in 1999/2000 to 19% in 2015/2016^42^. In U.S. construction, 54% of the workers with injuries received pain medications. Of those injured workers with pain medications, opioids were used in 47% and non-opioid medications in 35%. Thus, the overall pain medication use in injuries was comparable to our study, but the opioid use was substantially higher, which may still indicate a more restrictive use of opioids compared to the U.S.^43^.

The underlying reasons for different prescription practices are not well understood. The current study suggests that injury severity and type as well as injuries of the more proximal body parts and torso are associated with more pain medication use and overall opioid use. However, only few factors were related to strong opioid use (i.e., injury severity, fractures, superficial injuries, and injuries to the shoulders). The findings are not readily to explain and warrant further studies. Other and most likely individual preferences of physicians and patients may play a role. According to a study in Spanish primary care physicians, the personal experience was more important than guidelines^44^. Further, guideline awareness was associated with increased confidence in treating chronic non-cancer pain patients^45^. In a survey of family physicians, 72% consider chronic nonmalignant pain as an indication for opioid use^46^. Primary care physicians in Spain rated their certainty about the indication and how to use opioids as average 4–7 out of 10 suggesting a substantial amount of uncertainty^44^.

### Strength and limitations

In this study, we were able to assess in a representative cohort of Swiss workers with a wider range of MSK injuries the influence of patient characteristics, injury severity, and injury type. We carved a clear-cut view on the prescription practice depending on the injured body part and injury type. The sensitivity test underlines the robustness of these results. There are several limitations that need to be discussed. First, only invoices for outpatient health expenditures (e.g., from general practitioners, pharmacies, or hospital *out-*patients visits) were available. We had no billing information from in-hospital treatments. In-hospital tariffs are diagnosis related groups-based flat-rates without detailed information about the services that were provided. In-patients are more likely to be severe cases as compared to out-patients. Hospitals might give additional doses to in-patients upon their release so that pain treatment can be continued at home. Thus, we may underestimate the number of patients with additional pain medication use in particular in non-opioid pain medications. Second, treatment might have shifted over time between general practitioners, hospital out*-*patient and in-patient treatment and thus be associated with an increased need in outpatient pain medication prescription^47^. Third, injury types and injured body parts were based on claims-reports. Hence type of injury and body part may differ from the physicians’ diagnosis. Further, severity of the injury was based on the duration of sick-leave. Therefore, this definition for the severity of an injury used in this study may not reflect the true extent of injury. Finally, we had no clinical information available (e.g., obesity and comorbidities) and may be missing other potentially relevant variables (e.g., reimbursement of pain medications does not necessarily mean that patients also used the medication) which may explain variation in pain medication use. Thus, additional studies should explore practice variation in different areas with high and low pain medication use to better understand underlying mechanisms.

### Implication for clinical practice

Although opioid use, and in particular strong opioid use, in MSK injuries is not recommended as the first choice by guidelines, strong opioids are increasingly used^48^. General practitioners need to be aware of the potential unintended consequences especially when used in superficial and minor injuries. In Switzerland, general practitioners provide initial care in 56% of all injuries^47^. Between 2008 and 2014 the proportion of initial care that is provided in emergency departments increased from 38 to 46%. Thus, emergency department physicians also play a role in the increased use of strong opioids. With an increasing shortage of primary care physicians, awareness in guideline recommendations for the treatment of MSK injuries in emergency departments should be improved. Interventions to reduce the frequency of opioid prescriptions include increased awareness of prescribers and patients, drug monitoring programs, adapted remuneration systems, opioid education, and access to behavioral health services^49–53^. Physicians, patients, and policymakers should be informed about the potential unintended consequences of the use of strong opioids for MSK injuries observed in this study.

## Conclusion

In MSK injuries, strong opioids were increasingly used. Although the overall pain medication and the overall opioid use was higher in fractures, ruptures and injuries of the shoulder and chest, the increased use of strong opioids was also observed when controlling for injury type and body part, injury severity and other factors. This finding suggests that other factors, such as physicians’ preferences may be more important in the choice of strong opioids in MSK injuries. Future studies may identify the factors influencing the prescription behavior and help design programs to increase awareness of providers and patients about the benefit and risk of opioids.

### Supplementary Information

Supplementary Information 1.

Supplementary Information 2.

Supplementary Information 3.

## Data Availability

Due to privacy restriction, the data used for the current study cannot be made available. If there are further questions concerning the data sharing, contact Dr. Stefan Scholz, Suva, Department of Statistics, Lucerne, Switzerland, stefan.scholz@suva.ch.
