# Supplementary material for: Factors influencing pain medication and opioid use in patients with musculoskeletal injuries: a retrospective insurance claims database study
Source: Sci Rep. 2024 Jan 23;14:1978. doi: 10.1038/s41598-024-52477-7 (PMC10805862; doi:10.1038/s41598-024-52477-7)
Supplement: Supplementary file 1 — Supplementary Information 1. [file 41598_2024_52477_MOESM1_ESM.docx]

## **Appendix 1: Characteristics of injury type** × **body part included in the sensitivity analysis**

|  |  | **All injuries** | **Injuries with pain medication use** | **Injuries with opioid use** |
| --- | --- | --- | --- | --- |
|  |  |  | N (%) |  |
| Total |  | 4'124'755 (100) | 1'923'759 (100) | 227'317 (100) |
| injury | fracture × head | 20'240 (0.5) | 11'033 (0.6) | 1'143 (0.5) |
| type | fracture × lower torso | 7'202 (0.2) | 4'856 (0.3) | 1'816 (0.8) |
| × | fracture × upper torso | 49'559 (1.2) | 35'374 (1.8) | 14'392 (6.3) |
| injured | fracture × upper Extr | 172'596 (4.2) | 112'256 (5.8) | 23'680 (10.4) |
| body part | fracture × lower Extr | 124'662 (3.0) | 78'932 (4.1) | 13'496 (5.9) |
|  | sprain × head | 2'334 (0.1) | 1'080 (0.1) | 97 (0.0) |
|  | sprain × lower torso | 13'840 (0.3) | 7'117 (0.4) | 789 (0.3) |
|  | sprain × upper torso | 94'819 (2.3) | 51'937 (2.7) | 9'042 (4.0) |
|  | sprain × upper Extr | 210'302 (5.1) | 97'290 (5.1) | 11'633 (5.1) |
|  | sprain × lower Extr | 476'593 (11.6) | 238'685 (12.4) | 14'029 (6.2) |
|  | superficial × head | 77'748 (1.9) | 17'151 (0.9) | 777 (0.3) |
|  | superficial × lower torso | 4'742 (0.1) | 792 (0.0) | 66 (0.0) |
|  | superficial × upper torso | 8'775 (0.2) | 1'631 (0.1) | 193 (0.1) |
|  | superficial × upper Extr | 429'047 (10.4) | 122'832 (6.4) | 7'509 (3.3) |
|  | superficial × lower Extr | 99'365 (2.4) | 26'283 (1.4) | 1'394 (0.6) |
|  | contusion × head | 72'014 (1.7) | 27'235 (1.4) | 1'836 (0.8) |
|  | contusion × lower torso | 46'793 (1.1) | 25'970 (1.3) | 3'996 (1.8) |
|  | contusion × upper torso | 212'418 (5.1) | 128'952 (6.7) | 26'415 (11.6) |
|  | contusion × upper Extr | 386'687 (9.4) | 185'202 (9.6) | 16'511 (7.3) |
|  | contusion × lower Extr | 328'100 (8.0) | 157'544 (8.2) | 9'398 (4.1) |
|  | rupture × head | 30'343 (0.7) | 7'293 (0.4) | 242 (0.1) |
|  | rupture × lower torso | 4'408 (0.1) | 2'384 (0.1) | 413 (0.2) |
|  | rupture × upper torso | 10'903 (0.3) | 7'025 (0.4) | 1'755 (0.8) |
|  | rupture × upper Extr | 84'430 (2.0) | 42'932 (2.2) | 6'591 (2.9) |
|  | rupture × lower Extr | 175'295 (4.2) | 102'361 (5.3) | 8'601 (3.8) |
|  | other | 981'540 (23.8) | 429'612 (22.3) | 51'503 (22.7) |

Number of cases and column percentage

## **Appendix 2: Odds ratios for the models with and without interaction between injury type and body part (sensitivity analysis)**

|  |  | using all cases for predicting the  event “cases treated with any analgesic” | | using cases with analgesics for predicting the event “cases treated with opioid” | | using cases treated with opioids for predicting  the event “cases treated with strong opiod” | |
| --- | --- | --- | --- | --- | --- | --- | --- |
|  |  | model 1 | sensitivity analysis | model 2 | sensitivity analysis | model 3 | sensitivity analysis |
|  | Intercept | 1.350 (CI 1.325,1.375) | 1.428 (CI 1.417,1.440) | 0.096 (CI 0.092,0.100) | 0.189 (CI 0.186,0.192) | 0.344 (CI 0.312,0.379) | 0.263 (CI 0.252,0.274) |
| severity | minor (< 3 days lost) | 0.330 (CI 0.329,0.331) | 0.319 (CI 0.318,0.321) | 0.270 (CI 0.266,0.274) | 0.266 (CI 0.263,0.270) | 0.417 (CI 0.397,0.439) | 0.379 (CI 0.360,0.398) |
| year | 2008 | 0.863 (CI 0.855,0.872) | 0.861 (CI 0.853,0.870) | 0.926 (CI 0.906,0.948) | 0.924 (CI 0.904,0.945) | 0.843 (CI 0.798,0.891) | 0.844 (CI 0.799,0.892) |
|  | 2009 | 0.896 (CI 0.887,0.905) | 0.897 (CI 0.888,0.906) | 0.961 (CI 0.940,0.983) | 0.967 (CI 0.946,0.988) | 0.868 (CI 0.822,0.916) | 0.868 (CI 0.822,0.916) |
|  | 2010 | 0.945 (CI 0.936,0.954) | 0.945 (CI 0.936,0.954) | 1.002 (CI 0.980,1.024) | 1.004 (CI 0.983,1.026) | 0.881 (CI 0.835,0.929) | 0.879 (CI 0.834,0.926) |
|  | 2011 | 0.943 (CI 0.933,0.952) | 0.941 (CI 0.932,0.951) | 0.992 (CI 0.970,1.014) | 0.989 (CI 0.968,1.010) | 0.966 (CI 0.917,1.018) | 0.967 (CI 0.918,1.019) |
|  | 2012 | 0.979 (CI 0.970,0.989) | 0.978 (CI 0.968,0.987) | 0.988 (CI 0.966,1.010) | 0.986 (CI 0.965,1.008) | 0.961 (CI 0.912,1.012) | 0.968 (CI 0.919,1.020) |
|  | 2013 (ref) | 1.000 (CI 1.000,1.000) | 1.000 (CI 1.000,1.000) | 1.000 (CI 1.000,1.000) | 1.000 (CI 1.000,1.000) | 1.000 (CI 1.000,1.000) | 1.000 (CI 1.000,1.000) |
|  | 2014 | 1.029 (CI 1.019,1.040) | 1.027 (CI 1.017,1.038) | 0.978 (CI 0.957,0.999) | 0.974 (CI 0.953,0.996) | 1.086 (CI 1.032,1.144) | 1.090 (CI 1.036,1.147) |
|  | 2015 | 1.001 (CI 0.991,1.011) | 1.000 (CI 0.990,1.009) | 1.001 (CI 0.980,1.023) | 0.993 (CI 0.972,1.014) | 1.218 (CI 1.158,1.281) | 1.218 (CI 1.159,1.281) |
|  | 2016 | 0.982 (CI 0.973,0.992) | 0.981 (CI 0.971,0.990) | 0.991 (CI 0.969,1.013) | 0.983 (CI 0.962,1.004) | 1.278 (CI 1.216,1.344) | 1.280 (CI 1.218,1.346) |
|  | 2017 | 0.981 (CI 0.972,0.991) | 0.981 (CI 0.971,0.990) | 1.025 (CI 1.003,1.047) | 1.015 (CI 0.994,1.037) | 1.373 (CI 1.307,1.442) | 1.372 (CI 1.307,1.441) |
|  | 2018 | 0.919 (CI 0.910,0.928) | 0.920 (CI 0.911,0.929) | 0.996 (CI 0.975,1.018) | 0.990 (CI 0.969,1.011) | 1.503 (CI 1.431,1.578) | 1.506 (CI 1.435,1.580) |
| gender | female | 0.975 (CI 0.969,0.980) | 0.961 (CI 0.956,0.966) | 0.983 (CI 0.972,0.995) | 0.941 (CI 0.930,0.952) | 0.933 (CI 0.907,0.960) | 0.916 (CI 0.890,0.942) |
| age | 20-year point estimate | 0.925 (CI 0.922,0.928) | 0.901 (CI 0.899,0.904) | 0.751 (CI 0.746,0.756) | 0.706 (CI 0.701,0.711) | 1.163 (CI 1.143,1.183) | 1.156 (CI 1.137,1.176) |
|  | 30 | 0.962 (CI 0.960,0.963) | 0.949 (CI 0.948,0.951) | 0.867 (CI 0.863,0.870) | 0.840 (CI 0.837,0.843) | 1.079 (CI 1.069,1.088) | 1.075 (CI 1.066,1.084) |
|  | 40 (ref) | 1.000 (CI 1.000,1.000) | 1.000 (CI 1.000,1.000) | 1.000 (CI 1.000,1.000) | 1.000 (CI 1.000,1.000) | 1.000 (CI 1.000,1.000) | 1.000 (CI 1.000,1.000) |
|  | 50 | 1.040 (CI 1.038,1.041) | 1.053 (CI 1.052,1.055) | 1.154 (CI 1.150,1.158) | 1.190 (CI 1.186,1.194) | 0.927 (CI 0.919,0.935) | 0.930 (CI 0.922,0.938) |
|  | 60-year point estimate | 1.081 (CI 1.077,1.084) | 1.109 (CI 1.106,1.113) | 1.332 (CI 1.322,1.341) | 1.417 (CI 1.407,1.427) | 0.860 (CI 0.845,0.875) | 0.865 (CI 0.850,0.880) |

Odds ratios from logistic regression models and their confidence intervals (CI). Results are shown for models correcting for injury type and for the main models using body parts separately and for the sensitivity analysis correcting using a combined variable for interaction of injury type × body part. Odds ratios for these variables can be found in the Appendix Tables A and B, respectively. Point estimates are indicated for specific ages between 20 to 60 years.

**Appendix Figure 1: Estimates and Odds Ratios from appendix table 2 for pain medication, opioid, and strong opioids for the main models and the sensitivity analysis**

Estimates and odds ratios derived from logistic regression models using all cases for predicting the event “cases treated with any analgesic” (model 1, left), using cases with analgesics for predicting the event “cases treated with opioid” (model 2, center), and using cases treated with opioids for predicting the event “cases treated with strong opioid” (model 3, right). Main models with variables for injury type and body parts (red) and variant with combined variable for injury type × body part for sensitivity analysis (blue) are shown in each panel. Point estimates are indicated for specific ages between 20 to 60 years.

**Appendix Figure 2: Estimates and Odds Ratios for pain medication, opioid, and strong opioids for the sensitivity analysis by interacting injury type and body part**

Estimates derived from logistic regression models for combined injured body part × injury type variables from sensitivity analysis. Estimates and odds ratios derived from logistic regression models are shown using all cases for predicting the event “cases treated with any analgesic” (model 1, left), using cases with analgesics for predicting the event “cases treated with opioid” (model 2, center), and using cases treated with opioids for predicting the event “cases treated with strong opioid” (model 3, right).
