# Supplementary figures and images for: Factors influencing pain medication and opioid use in patients with musculoskeletal injuries: a retrospective insurance claims database study
Source: Sci Rep. 2024 Jan 23;14:1978. doi: 10.1038/s41598-024-52477-7 (PMC10805862; doi:10.1038/s41598-024-52477-7)

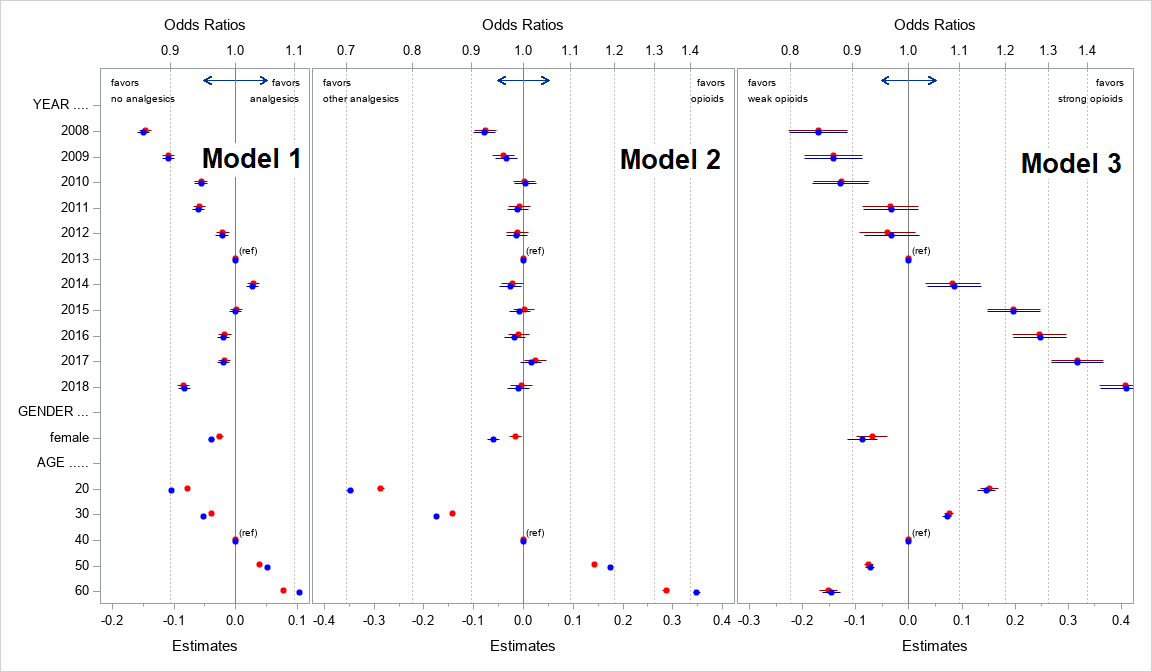

Supplement: Supplementary file 2 — Supplementary Information 2. [file 41598_2024_52477_MOESM2_ESM.png]

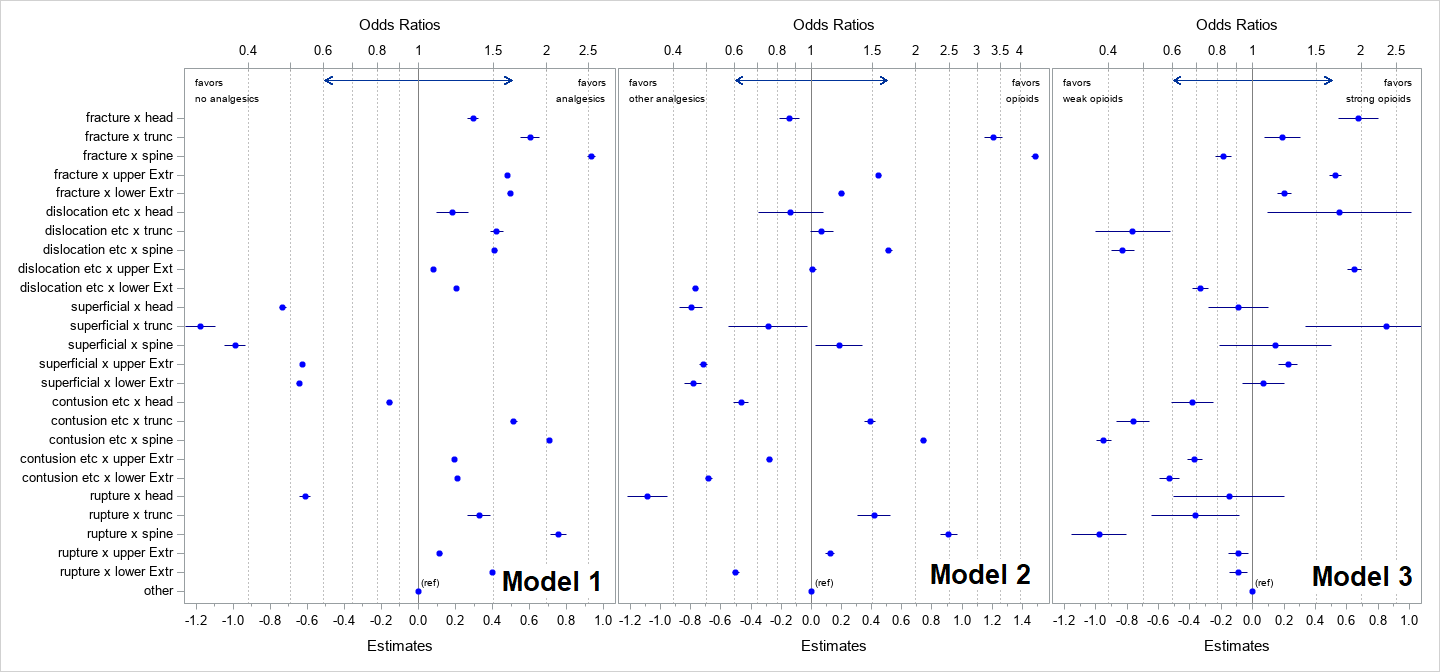

Supplement: Supplementary file 3 — Supplementary Information 3. [file 41598_2024_52477_MOESM3_ESM.png]
